# Optimization of Enzymatic Hydrolysis of *Perilla* Meal Protein for Hydrolysate with High Hydrolysis Degree and Antioxidant Activity

**DOI:** 10.3390/molecules27031079

**Published:** 2022-02-06

**Authors:** Henghui Zhang, Zhijun Zhang, Dongliang He, Shuying Li, Yongping Xu

**Affiliations:** 1 Department of Environment and Safety Engineering, Taiyuan Institute of Technology, Taiyuan 030008, China; hedl@tit.edu.cn; 2 School of Chemical Engineering and Technology, North University of China, Taiyuan 030051, China; 3 SEM Bio-Engineering Technology Co., Ltd., Dalian 116600, China; 4 School of Bioengineering, Dalian University of Technology, Dalian 116024, China; xyping@dlut.edu.cn

**Keywords:** *Perilla* meal protein, hydrolysate, response surface methodology, antioxidant activity, peptide

## Abstract

Botanical oils are staple consumer goods globally, but as a by-product of oil crops, meal is of low utilization value and prone to causing environmental problems. The development of proteins in meal into bioactive peptides, such as *Perilla* peptide, through biotechnology can not only solve environmental problems, but also create more valuable nutritional additives. In the present work, the hydrolysis process of *Perilla* meal protein suitable for industrial application was optimized with the response surface methodology (RSM) on the basis of single-factor experiments. Alcalase was firstly selected as the best-performing among four proteases. Then, based on Alcalase, the optimal hydrolysis conditions were as follows: enzyme concentration of 7%, hydrolysis temperature of 61.4 °C, liquid-solid ratio of 22.33:1 (mL/g) and hydrolysis time of 4 h. Under these conditions, the degree of hydrolysis (DH) of *Perilla* meal protein was 26.23 ± 0.83% and the DPPH scavenging capacity of hydrolysate was 94.15 ± 1.12%. The soluble peptide or protein concentration of *Perilla* meal protein hydrolysate rose up to 5.24 ± 0.05 mg/mL, the ideal yield of which was estimated to be 17.9%. SDS-PAGE indicated that a large proportion of new bands in hydrolysate with small molecular weights appeared, which was different from the original *Perilla* meal protein. The present data contributed to further, more specific research on the separation, purification and identification of antioxidant peptide from the hydrolysate of *Perilla* meal protein. The results showed that the hydrolysis of *Perilla* meal protein could yield peptides with high antioxidant activity and potential applications as natural antioxidants in the food industry.

## 1. Introduction

*Perilla frutescens* (L.) *Britt.* is an annual herb widely distributed in China, Japan, Korea, India, Myanmar, Russia and other countries, belonging to the genus *Perilla* in the *Labiaceae* family [1,2]. *Perilla*,which has been cultivated for more than 2000 years in China, is one of the first 60 medicinal and food homologous plants approved by the National Health Commission of the People’s Republic of China [3]. All parts of the *Perilla* plant have high utilization value; its dry leaves, stems and seeds are included in pharmacopoeia of China [4,5,6].

In previous studies, *Perilla* seeds were mainly used to obtain oils rich in unsaturated fatty acids for the food industry, resulting in the production of a large quantity of *Perilla* meal by-product [7]. Although *Perilla* meals are well-utilized, they also cause environmental problems [8]. However, the main component of defatted *Perilla* meal is protein, accounting for up to 65% of its content [9]. Moreover, *Perilla* meal is also a high-quality protein resource with complete amino acids and balanced composition, and the content of essential amino acids is similar to that of egg [10]. The low solubility and high molecular weight of *Perilla* meal protein limit its utilization [11]. In order to develop *Perilla* meal protein resources, the preparation of bioactive peptide by enzymatic hydrolysis of *Perilla* protein is one of the most feasible means [12].

Since their discovery in the 1920s, peptides and their effects on disease and aging continued to be studied in the following decades up to the 1990s [13]. During this time, a large number of bioactive peptides were isolated from plants, animals, and microorganisms [14]. In recent research, the preparation of bioactive peptide by the enzymatic hydrolysis method has been an area of interest [15]. Researchers have obtained peptides with different biological activities from soybean, rapeseed, sunflower seed, walnut and other plant proteins [16,17], as well as animal proteins such as *Antheraea pernyi* pupa, loach, grass carp, small tuna, oyster meat, etc. [18,19,20,21]. These peptides have been proved to possess antioxidant, blood pressure lowering, anti-bacterial, anti-tumor, immune regulation and anti-fatigue effects [18,20].

Oxidative stress is a negative effect caused by free radicals in the body and is considered to be an important cause of aging and disease [22,23]. The idea of obtaining antioxidative peptides by hydrolysis of *Perilla* meal protein could not only improve the value of *Perilla* meal, but also supply a safe and edible antioxidant to improve oxidative stress [24]. The aim of this study was to develop these *Perilla* meal waste proteins into value-added nutritional additives. The way to achieve that is to optimize the process for hydrolysis of *Perilla* meal protein by protease and evaluate the DH of protein and the antioxidant activity of hydrolysate. This work provided reliable data as a basis for the separation, purification and identification of antioxidant peptides through the hydrolysis of *Perilla* meal protein in further study.

## 2. Materials and Methods

### 2.1. Chemicals

Alcalase (2.4 AU/g) was obtained from Novozymes Biotech (Copenhagen, Denmark). Papain (8 × 10^5^ U/g), neutral protease (6 × 10^4^ U/g) and Flavourzyme (1.5 × 10^4^ U/g) were obtained from Beijing Solarbio Technology Co., Ltd. (Beijing, China). 2.2-diphenyl-1-picrylhydrazyl (DPPH) and N, N, N’, N’-tetramethylethylenediamine (TEMED) was obtained from Sigma-Aldrich (St. Louis, MO, USA). Copper sulphate, potassium sulphate, boracic acid, sulfuric acid, acrylamide, methylene diacrylamide, tris-(hydroxymethyl)-aminomethane (Tris), and lauryl sodium sulfate (SDS) were obtained from Sinopharm Chemical Reagents Co., Ltd. (Shanghai, China). Protein molecular weight marker (Low) was obtained from Takara Bio (Dalian) Co., Ltd. (Dalian, China). Coomassie brilliant blue R250, Coomassie brilliant blue G250, methyl red, methylene blue, ammonium persulfate and methanal and sodium hydroxide were obtained from Aladdin Reagent Co., Ltd. (Shanghai, China). All other chemicals used were of analytical grade.

### 2.2. Preparation of Defatted Perilla Meal Powder

*Perilla* seeds (Jin Perilla No. 1, Taiyuan, Shanxi Province, China) were collected from the experimental field of North University of China. After cleaning and pressing, *Perilla* meal was obtained from the seeds, and crushed into a powder using a high-speed pulverizer. Then, the powder was degreased by ether extraction and immediately dried to remove residual ether. Without sieving, the powder was stored under dry conditions and used as the starting material in the subsequent hydrolysis.

### 2.3. Determination of Protein Content in the Perilla Meal

According to Chinese national standard GB 5009.5-2016, the protein content of *Perilla* meal was determined by automatic Kjeldahl apparatus [25]. A *Perilla* meal sample of 1.0 g was digested at about 420 °C with 0.4 g of copper sulfate, 6.0 g of potassium sulfate and 20 mL of sulfuric acid until it appeared green and transparent. After cooling, 50 mL of deionized water was added to the mixture, which was then measured by automatic Kjeldahl apparatus. Following the calculation formula in the standard, the protein content was calculated on the basis of the measured results.

### 2.4. Enzymatic Hydrolysis of Perilla Meal Protein

The enzymatic hydrolysis of defatted *Perilla* meal protein was investigated using four different proteases: Alcalase, papain, neutral protease and flavourzyme [26]. A 10.0 g quantity of *Perilla* meal powder was added to deionized water according to a given liquid-solid ratio, and then the chosen protease was blended into the mixture on the basis of the set enzyme concentration. After fully stirring, the mixture was adjusted to the optimal pH value of the chosen protease, and then the enzymatic reaction was started in a thermostatic water bath [27]. After the scheduled time, the enzymatic hydrolysis of *Perilla* meal protein was terminated by boiling the mixture for 10 min until the protease was totally inactivated. Subsequently, to fix the new amino groups after breaking the peptide bonds, 40% formaldehyde solution was poured into the hydrolysate, and 0.1 M NaOH solution was immediately added dropwise into the mixture to reach the initial pH value. Then, the amount of added NaOH solution used was recorded, and the hydrolysate was centrifuged to obtain the supernatant, which was stored at −20 °C until further use [28].

### 2.5. Determination of Degree of Hydrolysis

The degree of hydrolysis of *Perilla* protein hydrolysate was measured by pH-stat method with slight modifications [29,30]. Free amino and carboxyl groups were formed after hydrolysis of peptide bonds, and the dissociation states of the two groups varied under different pH conditions. When pH > 6, the amount of H^+^ released by the hydrolysate was greater than the amount of H^+^ bound; we thus fixed the free amino groups by adding 40% formaldehyde solution to dissociate the free carboxyl groups and lower the pH [31,32]. In order to keep pH the same as the original value, NaOH solution needed to be added to the mixture. So, the consumption of NaOH solution was proportional to the number of carboxyl groups produced after the reaction, as well as hydrolyzed peptide bonds and DH of the hydrolysate [33]. The DH of the hydrolysate was calculated by the equation given below:

(1) $$\mathrm{DH}(\%)=\frac{h}{h_{\mathrm{tot}}}\times 100=\frac{V\times N}{\alpha \times M_{p}\times h_{\mathrm{tot}}}\times 100$$

where h represents the amount of hydrolyzed peptide bonds per unit mass of protein (mmol/g); h_tot_ represents the total amount of peptide bonds per unit mass of protein (mmol/g) (the h_tot_ of *Perilla* protein was 8.0); V represents the consumed volume of NaOH solution (mL); N represents the molarity of NaOH solution (mol/L); α represents the dissociation degree of α-amino acid (α could be 0.986 here); and M_p_ represents the total mass of protein in the sample.

### 2.6. Evaluation of Antioxidant Activity

In recent years, many methods have been used to estimate the antioxidative activity of antioxidant candidates [34]. Free radical scavenging activities are often used to evaluate antioxidant activities in vitro [35]. In this work, the assay of DPPH· radical scavenging activity was selected for the rapid assessment of the potential antioxidant activity of the hydrolysate of *Perilla* meal protein. The DPPH· radical scavenging capacity of the hydrolysate of *Perilla* meal protein was tested based on the method reported by Xu with slight modification [36]. Briefly, 0.1 mL of the liquid supernatant of hydrolysate was mixed with 0.9 mL of the DPPH solution (60 μmol/L soluble in ethanol). After blending, the mixture was placed in darkness for 30 min. Then, absorbance of the mixture at 518 nm was recorded immediately with ethanol as a reference [37]. The percentage of DPPH· radical scavenging was calculated using the following equation:

(2) $$\mathrm{DPPH}\;\mathrm{radical}\;\mathrm{scavenging}\;\mathrm{activity}\;(\%)=(1-\frac{A_{S}-A_{r}}{A_{0}})\times 100$$

where A_s_ represents the absorbance of sample solution after reacting with DPPH solution, A_r_ represents the absorbance of sample solution with reference (absolute ethanol), and A_0_ represents the absorbance of ultrapure water with DPPH solution. Each test was repeated three times.

### 2.7. Optimization of Enzymatic Hydrolysis of Perilla Meal Protein

#### 2.7.1. Single-Factor Experiments

Prior to the hydrolysis experiments, the optimum protease was studied initially by comparing four candidates, respectively Alcalase, papain, neutral protease and flavourzyme according to the evaluation of DH and DPPH· radical scavenging ability of hydrolysate. Then, using the DH and DPPH· radical scavenging ability as measurements, four factors were selected and studied as independent variables, including which were liquid-solid ratio, enzyme concentration, hydrolytic temperature and hydrolytic time [20]. First, to study the impact of the liquid-solid ratio on the two indexes, 10.0 g of the *Perilla* meal powders were soaked in deionized water with different liquid-solid ratios (10:1, 12.5:1, 20:1, 50:1, mL/g), the enzyme concentration was set at 5% (*g*/*g*), and the hydrolytic temperature and time were set at 60 °C and 4 h, respectively. Then, to investigate the effect of enzyme concentration, different enzyme concentrations (1%, 3%, 5%, 9%) were prepared under the conditions of liquid-solid ratio 20:1, hydrolytic temperature 60 °C and hydrolytic time 4 h. Subsequently, multiple hydrolytic temperatures (50 °C, 55 °C, 60°C and 65 °C) were tested under the conditions of liquid-solid ratio 20:1, enzyme concentration 5%, and hydrolytic time 4 h to evaluate the effect of hydrolytic temperature on the two indexes. Finally, to choose the optimum hydrolytic time, different hydrolytic times (0.5, 1.0, 2.0, 4.0, 6.0, 8.0 and 10.0 h) were studied under the following conditions: liquid-solid ratio 20:1, enzyme concentration 5%, and hydrolytic temperature 60 °C [16]. At the end of each hydrolysis reaction, the supernatant was collected after centrifugation (5000 rpm, 4 min) and set aside for further analysis.

#### 2.7.2. Response Surface Methodology (RSM) Design

On the basis of single-factor experimental results, the enzymatic hydrolysis of *Perilla* meal protein was optimized by response surface methodology (RSM). In this study, the Box–Behnken design method (BBD) provided by Design Expert 11 was selected to guide the experimental design with three of the four variables chosen from the single-factor experiments at three levels, whose response values were the DH and DPPH· radical scavenging ability of hydrolysate [38]. The BBD method with three factors and three levels consisted of 17 randomized experiments.

According to the experimental results of each trial, each quadratic equation model was fitted to calculate each response value, respectively, including the DH and DPPH· radical scavenging ability, and to assess the importance of the factors and their interactions in the enzymatic hydrolysis process [39]. The design of the experiment is shown in Table 1.

### 2.8. Evaluation of Soluble Protein Concentration

The water solubility of most proteins in meal is poor, which is also the main reason why protein can not be utilized efficiently. After hydrolysis, the insoluble protein of *Perilla* meal was broken down into peptides or proteins with smaller molecular weights, and the solubility of hydrolysis increased. The content of soluble protein or peptide in the hydrolysate could also reflect the DH of the *Perilla* meal protein.

The soluble protein concentration was measured by the classic Bradford dye method. When the protein was combined with Coomassie brilliant blue G250 dye, the conjugate had a maximum light absorption value at 595 nm. Additionally, the light absorption was proportional to the protein content. According to the linear equation fitted by the standard curve, the absorbance value at 595 nm of the sample could be calculated relative to concentration [40].

### 2.9. SDS-PAGE

The molecular weights of proteins could be directly observed by SDS-PAGE. By observing and comparing the SDS-PAGE results of the samples before and after hydrolysis, we could observe the changes induced by protease on the meal protein. In this work, we chose reduced SDS-PAGE of 12.5% separated glue to study the changes in protein molecular weight in the hydrolysate [41]. *Perilla* protein isolate and its three components, *Perilla* globulins, *Perilla* albumins, and *Perilla* glutens, were selected as controls.

### 2.10. Statistical Analysis

All experiments were independently repeated at least three times, and data were expressed as means ± SD for each experiment. A variance analysis (ANOVA) was used and differences in average DH and DPPH· radical scavenging ability were analyzed by *F*-test. The results of RSM were tested using Design-Expert 11 (Stat-Ease Inc., Minneapolis, MN, USA). The DH and DPPH· radical scavenging ability of the hydrolysate were assessed with the GraphPad Prism 8.0 program (GraphPad Software Inc., La Jolla, CA, USA). *p* ≤ 0.05 was considered statistically significant.

## 3. Results and Discussion

### 3.1. The Content of Protein in the Perilla Meal

The crude protein content in the defatted *Perilla* meal was determined by the Kjeldahl method as 65.17 ± 0.15%. The results showed that the protein content in *Perilla* meal was higher than that in sunflower meal (62.22%) and rapeseed meal (38.40%), which indicated that *Perilla* meal was a good protein source with high utilization value and development potential.

### 3.2. Selection of the Optimal Protease

**Figure 1:**
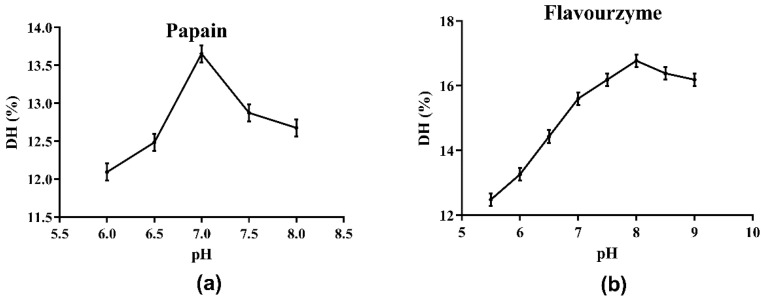
Determination of optimal pH of hydrolysis by proteases: (**a**) papain; (**b**) flavourzyme.

**Figure 2:**
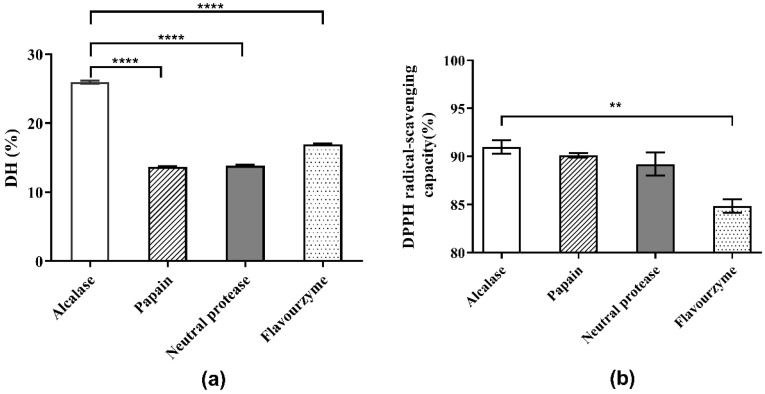
Comparison of hydrolysis and antioxidant activities of four proteases: (**a**) DH of *Perilla* meal protein after hydrolysis; (**b**) DPPH scavenging capacity of hydrolysates. **** means extreme significance of the difference (*p* < 0.0001). ** means high significance of the difference (*p* < 0.01).

The choice of protease directly affects the results of the enzymatic hydrolysis reaction and the properties of the enzymatic hydrolysate, including the molecular weight of peptides or proteins, the DH of hydrolysate, and the biological activities of the generated peptides [16]. Initially, the optimum pH values of the four protease candidates were determined. As shown in Figure 1 and the supplied protease directions, the optimum pH values for Alcalase, papain, neutral protease and flavourzyme were determined as 8.0, 7.0, 7.0, and 8.0, respectively. The effects of the four protease candidates on DH and DPPH· radical scavenging ability are shown in Figure 2. As shown, the DH of the hydrolysate using Alcalase, which hydrolyzed about 25.94% of the total meal protein, was obviously higher than the values obtained with the other three proteases. Furthermore, the DPPH· radical scavenging ability of the hydrolysate obtained with Alcalase was 91.01%, the highest among the four proteases. Therefore, Alcalase was selected as the optimal protease in later experiments.

### 3.3. Single-Factor Experiments to Assess DH and DPPH· Radical Scavenging Ability

#### 3.3.1. Effect of Liquid-Solid Ratio

**Figure 3:**
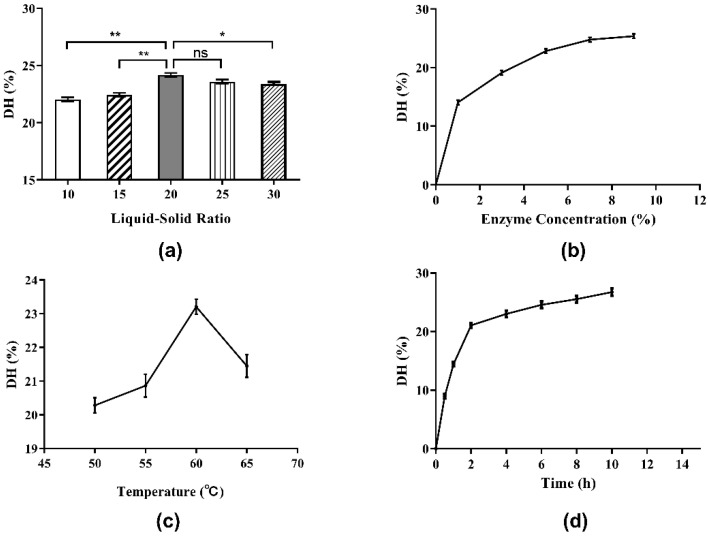
Single-factor experiment results for the DH of *Perilla* meal protein: (**a**) effect of liquid-solid ratio on the DH; (**b**) effect of enzyme concentration on the DH; (**c**) effect of hydrolysis temperature on the DH; (**d**) effect of hydrolysis time on the DH. The results are shown as mean ± SD. ** means high significance of the difference (*p* < 0.01). * means significance of the difference (*p* < 0.05). “ns” means no significance.

It is very important to select the proper liquid–solid ratio for the hydrolysis of *Perilla* protein to yield peptides in industrial production because it is a matter of cost and benefit. The influence of the liquid-solid ratio on DH of *Perilla* meal protein is shown in Figure 3a. As shown, the DH of *Perilla* meal protein rose when the liquid-solid ratio increased from 10:1 to 20:1. However, when the liquid-solid ratio exceeded 20:1, the DH of *Perilla* meal protein began to decline as the ratio continued to increase. According to the Michaelis–Menten equation given below, when the liquid-solid ratio was relatively low, the soluble components of the liquid including enzymes and substrates were almost saturated, that is, the enzymatic hydrolysis rate (v_0_) reached its maximum rate (V_max_). Therefore, the actual rate of enzymatic reaction (v_0_) as well as the maximum hydrolysis reaction rate (V_max_) increased with the increase in liquid-solid ratio. Hence, the DH of protein increased as well. When the liquid-solid ratio increased to a certain extent, the substrate concentration (S) started to decline as the ratio increased, leading to a decrease in the enzymatic hydrolysis rate (v_0_) and the DH of protein.

(3) $$v_{o}=\frac{V_{\max}\left[S\right]}{K_{m}+\left[S\right]}$$

where v_0_ represents the actual rate of enzymatic reaction, V_max_ represents the maximum rate of enzymatic reaction under certain conditions, K_m_ represents the Michaelis constant under those conditions, and [S] represents the substrate concentration.

**Figure 4:**
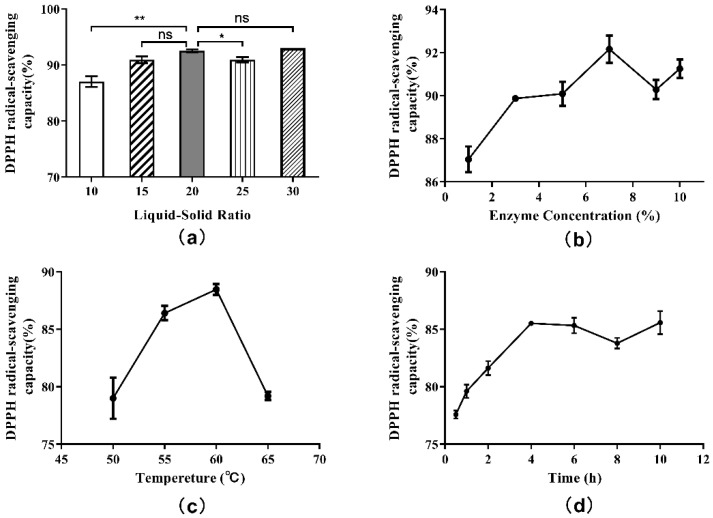
Single-factor experiment results for the DPPH radical scavenging capacity of hydrolysate generated from *Perilla* meal protein: (**a**) effect of liquid-solid ratio; (**b**) effect of enzyme concentration; (**c**) effect of hydrolysis temperature; (**d**) effect of hydrolysis time. The results are shown as mean ± SD. ** means high significance of the difference (*p* < 0.01). * means significance of the difference (*p* < 0.05). “ns” means no significance.

According to the single-factor experiment protocol, hydrolysates obtained from each experimental group were centrifuged at 7000× *g* for 4 min, and the supernatants were collected. Then, the antioxidant activities of supernatants were evaluated. Additionally, measurements of DPPH free radical scavenging capacities were included in the evaluation. The effect of liquid-solid ratio on the DPPH· radical scavenging ability of hydrolysate is shown in Figure 4a. The antioxidant activities of supernatants were found to increase with increasing liquid-solid ratios, reaching a maximum at 20:1, but started to decrease at ratios approaching 25:1. When the liquid-solid ratio was 30:1, there was a slight rebound in antioxidant activity. This trend indicated that the antioxidant activity of the hydrolysate was related not only to the degree of hydrolysis, but also to the length of peptide and amino acid sequences. Thus, the 20:1 liquid-solid ratio in the single factor experiment was selected as the appropriate ratio after comprehensive consideration.

#### 3.3.2. Effect of Enzyme Concentration

Enzyme concentration is undoubtedly a remarkable factor in the enzymatic hydrolysis reaction. As shown in Figure 3b, the DH clearly rose at the beginning when enzyme concentration increased from 1% to 7%. Then, the DH of protein increased slowly with increments of enzyme concentration beyond 7%. Eventually, as the enzyme concentration continued to increase, the DH tended to stabilize. These results could also be explained by the Michaelis–Menten equation. When the enzyme concentration was low and the enzyme could dissolve completely, the enzymatic hydrolysis rate (v_0_) and the DH of protein increased rapidly with the increase in enzyme concentration as well as the maximum hydrolysis reaction rate (V_max_). Until the enzyme concentration became saturated, the maximum rate of enzymatic reaction (V_max_) tended to be stable.

In this case, the actual rate of enzymatic reaction (v_0_) and the DH also remained stable, and the effect of increasing enzyme concentration on the DH of protein was no longer obvious. The influence of enzyme concentration on the DPPH· radical scavenging ability was determined by testing the hydrolysate supernatants above (Figure 4b). The antioxidant activities of supernatants were found to increase when the enzyme concentration was increased beyond 7%. Subsequently, with the increase in enzyme dosage, the antioxidant activity showed a wave trend, indicating that the correlation between antioxidant activity and enzyme concentration was not completely positive. The antioxidant activity as well as the DH were highest when the enzyme concentration was 7%. Therefore, 7% was chosen as the proper enzyme concentration.

#### 3.3.3. Effect of Hydrolysis Temperature

In this work, the influence of diverse hydrolysis temperatures on the DH of protein was studied, and the results are shown in Figure 3c. Temperature had a significant effect on enzymatic hydrolysis. When the hydrolysis temperature was low, the DH of protein increased with the increase in temperature. After reaching a maximum of about 23% at the hydrolysis temperature of 60 °C, the DH of protein exhibited a clear downward trend with further increases in temperature. The possible reason for this result was the duality of temperature to enzymatic reactions. On the one hand, as the temperature rose, the molecular movement accelerated and the chemical reaction rate increased. On the other hand, the increase in temperature would gradually denature and deactivate the protease, and its catalytic capacity would decline, resulting in a decrease in the hydrolysis reaction rate.

The effect of hydrolysis temperature on the DPPH· radical scavenging ability is shown in Figure 4c. As shown, the influence of hydrolysis temperature on the DPPH· radical scavenging ability was similar to the influence of temperature on the degree of hydrolysis. The highest DPPH· free radical scavenging rate was obtained when the hydrolysis temperature was 60 °C, which was also consistent with other reports. In consideration of DH and antioxidant activity, 60 °C was regarded as the best hydrolysis temperature.

#### 3.3.4. Effect of Hydrolysis Time

Figure 3d depicts the influence of hydrolysis time (0.5 to 10.0 h) on the DH. At first, the DH of protein improved quickly as the extraction time (from 0.5 to 2.0 h) was extended. However, when the extraction time increased to 2.0 h, the rate of increase in DH slowed down. In brief, the prolongation of hydrolysis time is beneficial to the hydrolysis process. With the passage of hydrolysis time, the substrate concentration decreased and the hydrolysis of protein gradually approached its end after 8 h.

The effect of hydrolysis on the DPPH· radical scavenging ability over time is plotted in Figure 4d. In the early stage of enzymatic hydrolysis, the DPPH· radical scavenging ability of hydrolysate supernatant increased with time and DH, and the antioxidant activity of the product reached a peak at the 4 h time point. Between 4 and 8 h of hydrolysis, the antioxidant activity of the hydrolysate began to decrease. After 8 h, the antioxidant activity increased again. These results once again indicated that the antioxidant activity of the hydrolysates was associated with the length and structure of peptide, amino acid sequence and exposed terminal amino acid residues. Hence, to consider the hydrolysis effect, time cost and antioxidant activity comprehensively, the optimal hydrolysis time was set at 4 h.

### 3.4. Optimization of Enzymatic Hydrolysis with DH as Response Value by RSM

#### 3.4.1. Model Fitting and Statistical Analysis

Through the analysis of single-factor experimental results, three variables (liquid-solid ratio, enzyme concentration and hydrolytic temperature) were used in the response surface methodology experiments. According to the BBD method, the experimental data on RSM with DH as response values are given in Table 2.

The RSM test results for the DH of *Perilla* meal protein were analyzed by statistical regression, and a quadratic polynomial regression equation was fitted as below:

(4) $$Y_{1}=26.18+0.45\times A+1.87\times B+0.22\times C-0.52\times A^{2}-3.00\times B^{2}-0.60\times C^{2}\;-0.42\times A\times B-0.098\times A\times C+0.83\times B\times C$$

where A, B, and C are the enzyme concentration, hydrolysis temperature and liquid-solid ratio, respectively. Y_1_ is the predicted value of DH.

The fitness of the regression model was evaluated by the method of ANOVA. The results of analysis are shown in Table 3. The F-value and p-value of the regression model were 14.34 and 0.001, respectively. The values indicated the model fit well. The lack of fit was not significant. That meant no additional optimization experiments were needed. The R^2^ and adjusted R^2^ of the model were 0.9785 and 0.9424, respectively, which showed that the model could fit the variability of response values well. Comparing F values showed that B (hydrolysis temperature) had the greatest influence on the DH, followed by A (enzyme concentration) and C (liquid-solid ratio). The linear term B and quadratic term B^2^ had extremely significant effects on the DH (*p* < 0.001).

#### 3.4.2. Analysis of Interaction between Factors

**Figure 5:**
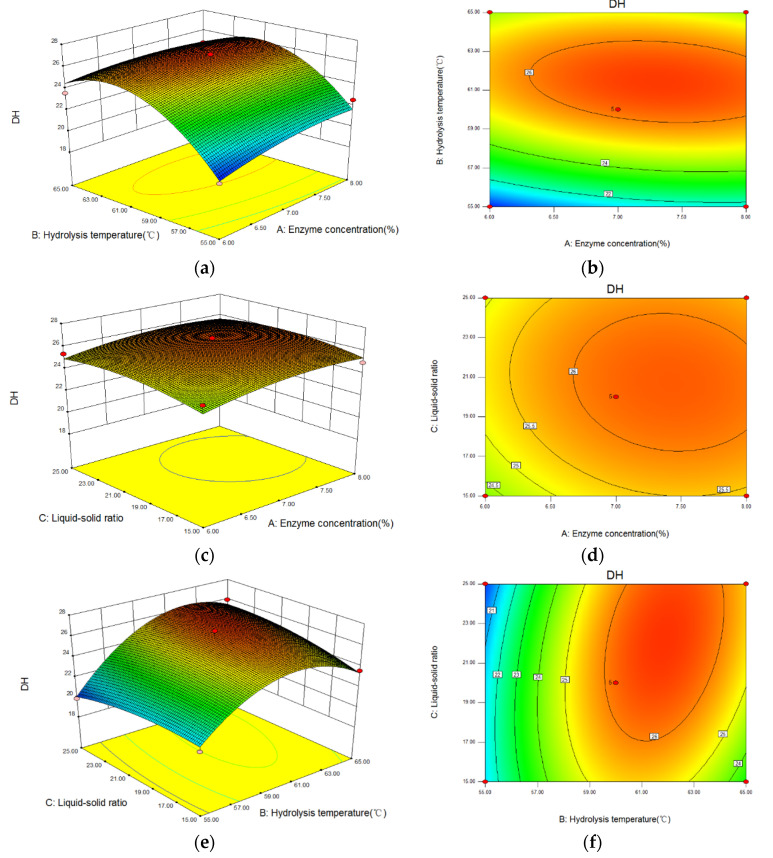
The interaction between variables on the DH of *Perilla* meal protein shown by response surface and contour plots. (**a**,**b**): the response surface and contour plots of enzyme concentration and hydrolysis temperature; (**c**,**d**): the plots of enzyme concentration and liquid-solid ratio; (**e**,**f**): the plots of hydrolysis temperature and liquid-solid ratio.

The 3D response surface and contour images of the interaction of A (enzyme concentration), B (hydrolysis temperature), and C (liquid-solid ratio) are given in Figure 5. As shown, hydrolysis temperature had the greatest influence on the DH because of the steep curve. The ovality of the contour plot formed by the interaction between enzyme concentration and hydrolysis temperature was the largest; that between hydrolysis temperature and liquid-solid ratio was second, and that between enzyme concentration and liquid-solid ratio was the smallest. Therefore, the influence of the interaction between enzyme concentration and hydrolysis temperature on the DH was the most significant.

### 3.5. Optimization of Enzymatic Hydrolysis with DPPH· Scavenging Capacity as Response Value by RSM

#### 3.5.1. Model Fitting and Statistical Analysis

The experimental results from the BBD method with DPPH· scavenging capacity as the response value are shown in Table 4.

The RSM test results for the DPPH· scavenging capacity of hydrolysate were analyzed by statistical regression, and a quadratic polynomial regression equation was fitted as below:

(5) $$Y_{2}=94.27-0.48\times A+0.88\times B+0.11\times C-1.46A^{2}-3.50\times B^{2}\;\;-0.32\times C^{2}-0.52\times A\times B+0.77\times A\times C+1.04\times B\times C$$

where A, B, and C are the enzyme concentration, hydrolysis temperature, and liquid-solid ratio, respectively. Y_2_ is the predicted value of the DPPH· scavenging rate.

The fitness of the regression model was evaluated by ANOVA. The results of analysis are shown in Table 5. The F-value and *p*-value of the regression model were 80.27 and 0.0217, respectively. The values indicated the model fit well; the lack of fit was not significant. That meant no further optimization experiments were needed. The R^2^ and adjusted R^2^ of the model were 0.9373 and 0.9424, respectively, which showed that the model could fit the variability of response values well. Comparing F values showed that B (hydrolysis temperature) had the greatest influence on the DPPH· scavenging capacity, followed by A (enzyme concentration) and C (liquid-solid ratio). The quadratic term B^2^ had an extremely significant effect on the DPPH· scavenging capacity (*p* < 0.001).

#### 3.5.2. Analysis of Interaction between Factors

**Figure 6:**
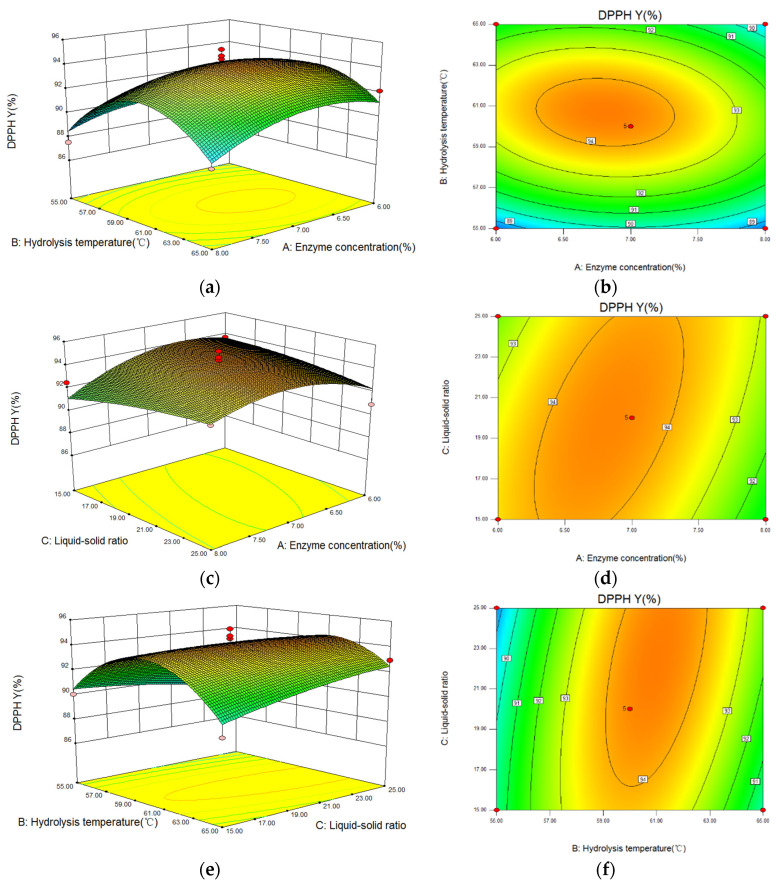
The influence of the interaction between variables on the DPPH radical scavenging capacity of hydrolysate generated from *Perilla* meal protein shown by response surface and contour plots. (**a**,**b**): the response surface and contour plots of enzyme concentration and hydrolysis temperature; (**c**,**d**): the plots of enzyme concentration and liquid-solid ratio; (**e**,**f**): the plots of hydrolysis temperature and liquid-solid ratio.

The three-dimensional (3D) response surface and contour images of the interaction of A (enzyme concentration), B (hydrolysis temperature), and C (liquid-solid ratio) are given in Figure 6. As shown, hydrolysis temperature had the greatest influence on the DPPH· scavenging capacity because of the steep curve. The ovality of the contour plot formed by the interaction between hydrolysis temperature and liquid-solid ratio was the largest, while that between enzyme concentration and liquid-solid ratio was the second-largest, and the one between enzyme concentration and hydrolysis temperature was the smallest. Therefore, the influence of the interaction between hydrolysis temperature and liquid-solid ratio on DPPH· scavenging capacity was the most significant.

### 3.6. Optimal Solution and Validation with Two Response Values

In the optimization of experiments by RSM with two response values, desirability stands for the comprehensive evaluation of two models whose values range from 0 to 1. According to the analysis in Design-Expert 11, the optimal solution of hydrolysis was predicted as follows: enzyme concentration was 7%, hydrolysis temperature was 61.38 °C and liquid-solid ratio was 22.33:1. Under these conditions, the theoretical DH of *Perilla* meal protein was 26.54%, and the DPPH· scavenging capacity of hydrolysate was 94.36%. These optimal conditions were modified as follows: enzyme concentration 7%, hydrolysis temperature 61.4 °C, and liquid-solid ratio 22.33:1 (mL/g). Subsequently, confirmatory experiments were arranged under the above conditions to verify the accuracy of the prediction models. The results with repeated tests showed that the DH was 26.23 ± 0.83% and the DPPH· scavenging capacity was 94.15 ± 1.12%, which were close to the theoretical values. The results indicated that the regression models fitted well and could be used to optimize the enzymatic hydrolysis process of *Perilla* meal protein.

### 3.7. Determination of Soluble Protein/Peptide Concentration in the Hydrolysate

*Perilla* meal protein was almost insoluble, and after hydrolysis, the solubility of the hydrolysate increased. According to the absorbance value of the sample obtained under the optimal conditions, the soluble peptide or protein concentration of the hydrolysate was calculated by the protein standard curve as 5.24 ± 0.05 mg/mL. Based on the used liquid-solid ratio, the ideal yield of soluble peptide or protein from *Perilla* meal after hydrolysis was estimated to be 17.9%.

### 3.8. SDS-PAGE Results for the Hydrolysate

**Figure 7:**
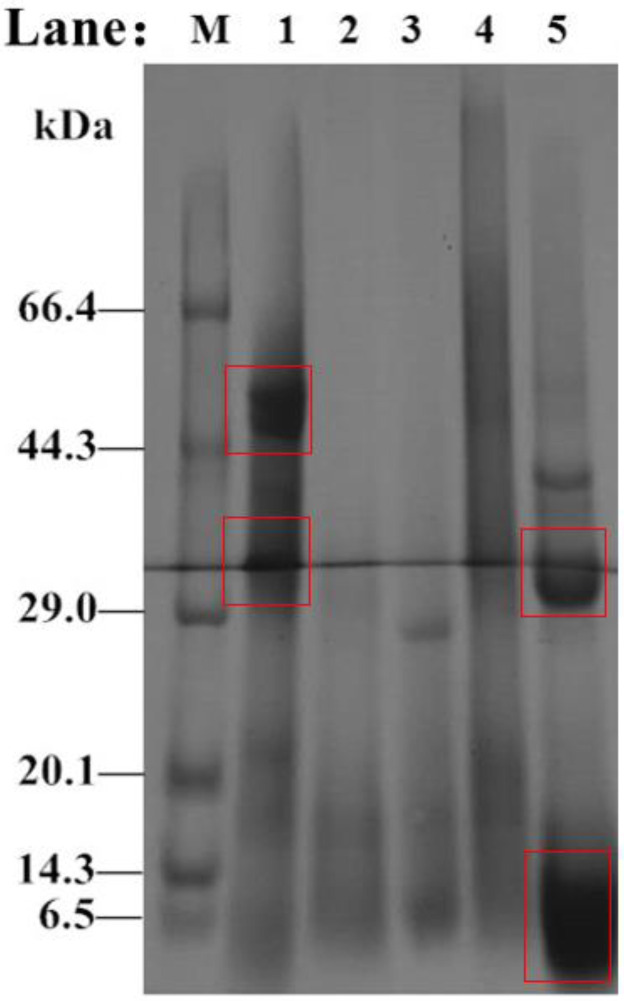
SDS-PAGE results for proteins isolated from *Perilla* meal and hydrolysate. **M**: Protein low MW markers; **Lane 1**: *Perilla* protein isolate; **Lane 2**: *Perilla* globulins; **Lane 3**: *Perilla* albumins; **Lane 4**: *Perilla* glutens; **Lane 5**: crude hydrolysate.

The molecular weight changes in *Perilla* meal protein before and after hydrolysis are shown in the SDS-PAGE results (Figure 7). As shown in Figure 7 Lane 1, there were two main bands of *Perilla* protein isolate in *Perilla* meal. One had a molecular weight of about 52 kDa, and the other had a molecular weight of about 32 kDa. Besides, there were few bands with molecular weight less than 6.5 kDa. However, unlike the bands before hydrolysis, the SDS-PAGE results for hydrolysate of *Perilla* meal protein were mainly composed of bands of less than 10 kDa, or even smaller molecular weights. Through analysis and judgment, soluble protein fragments and peptides with small molecular weights obtained by hydrolysis should exist in this band. In addition, a small number of unhydrolyzed or hydrolyzed proteins with larger molecular weights existed in the 37 kDa and 30 kDa bands. Thus, the appearance of new bands was the result of protease digestion. Meanwhile, the digestion of protein by protease, i.e., DH could not reach 100% like a strong acid or strong base.

## 4. Conclusions

In this study, we explored the enzymatic hydrolysis of *Perilla* meal protein to produce antioxidant peptides. Using DH and antioxidant activity as dual indexes, single-factor tests and RSM were conducted to optimize the hydrolysis conditions suitable for industrial applications. The models could be used to fit the hydrolytic process and predict the optimal conditions for hydrolysis of *Perilla* meal protein. The optimal conditions for hydrolysis of *Perilla* meal protein were modified as follows: enzyme concentration of 7%, hydrolysis temperature of 61.4 °C, liquid-solid ratio of 22.33:1 (mL/g), and hydrolysis time of 4 h. Under these conditions, the DH of *Perilla* meal protein was 26.23 ± 0.83%, and the DPPH· scavenging capacity of hydrolysate was 94.15 ± 1.12%. Moreover, after hydrolysis, the soluble peptide or protein concentration in *Perilla* meal protein rose to 5.24 ± 0.05 mg/mL. Based on these data, the ideal yield of soluble peptide or protein from *Perilla* meal after hydrolysis was estimated to be 17.9%. It was also directly proved by SDS-PAGE that a large proportion of new bands in hydrolysate with small molecular weights appeared, which was different from the original *Perilla* meal protein. In following work, we will separate and purify peptides with high antioxidant activity from the hydrolysate and determine the amino acid sequence. In addition, more in vitro and in vitro studies of *Perilla* peptides will be conducted to evaluate their potential as nutritional additives [42]. In summary, the present work should contribute to further, more specific research on the separation, purification and identification of antioxidant peptides by hydrolyzing *Perilla* meal protein, which might be used as a novel source of natural antioxidants in the food industry.

## Figures and Tables

**Table 1:** Levels and factors arrangement of response surface design.

Factors	Levels		
	−1	0	1
A: Enzyme concentration	6%	7%	8%
B: Hydrolysis temperature	55	60	65
C: Liquid-solid ratio	15	20	25

**Table 2:** Design and results of RSM with DH as response value.

No.	A: Enzyme Concentration	B: Hydrolysis Temperature	C: Liquid-Solid Ratio	Response 1 DH
	(%)	(°C)		(%)
1	8.00	60.00	25.00	24.96
2	6.00	60.00	15.00	24.96
3	7.00	6s0.00	20.00	26.92
4	7.00	60.00	20.00	25.94
5	6.00	55.00	20.00	19.70
6	8.00	60.00	15.00	24.96
7	7.00	65.00	25.00	25.94
8	6.00	60.00	25.00	25.35
9	7.00	65.00	15.00	23.60
10	7.00	55.00	25.00	19.89
11	7.00	60.00	20.00	26.14
12	6.00	65.00	20.00	23.60
13	7.00	55.00	15.00	20.87
14	8.00	65.00	20.00	24.77
15	7.00	60.00	20.00	25.94
16	7.00	60.00	20.00	25.94
17	8.00	55.00	20.00	22.53

**Table 3:** ANOVA for RSM quadratic model with DH as response value.

Source	Sum of Squares	Df	Mean Square	*F*-Value	*p*-Value	Significance
Model	75.93	9	8.44	14.34	0.0010	***
A	1.63	1	1.63	2.77	0.1401	
B	27.83	1	27.83	47.28	0.0002	***
C	0.38	1	0.38	0.65	0.4465	
A^2^	1.15	1	1.15	1.95	0.2055	
B^2^	38.00	1	38.00	64.57	0.0001	***
C^2^	1.50	1	1.50	2.55	0.1545	
AB	0.69	1	0.690	1.170	0.3151	
AC	0.038	1	0.038	0.065	0.8067	
BC	2.76	1	2.760	4.680	0.0672	
Residual	4.12	7	0.59			
Lack of fit	3.40	3	1.13	6.28	0.0541	
Pure error	0.72	4	0.18			
Cor total	80.05	16				
R ^2^ = 0.9785						
R ^2^_Adj_ = 0.9424						

*** means extreme significance of the difference (*p* < 0.001). Df means degree of freedom.

**Table 4:** Design and results of RSM with DPPH scavenging ability as response value.

No.	A: Enzyme Concentration	B: Hydrolysis Temperature	C: Liquid-Solid Ratio	Response 2 DPPH Scavenging Ability
	(%)	(°C)		(%)
1	8.00	60.00	25.00	92.84
2	6.00	60.00	15.00	93.68
3	7.00	60.00	20.00	92.68
4	7.00	60.00	20.00	95.33
5	6.00	55.00	20.00	88.80
6	8.00	60.00	15.00	92.52
7	7.00	65.00	25.00	92.94
8	6.00	60.00	25.00	90.94
9	7.00	65.00	15.00	89.21
10	7.00	55.00	25.00	89.63
11	7.00	60.00	20.00	94.56
12	6.00	65.00	20.00	92.12
13	7.00	55.00	15.00	90.04
14	8.00	65.00	20.00	88.80
15	7.00	60.00	20.00	94.00
16	7.00	60.00	20.00	94.80
17	8.00	55.00	20.00	87.55

**Table 5:** ANOVA for RSM quadratic model with DPPH scavenging ability as response value.

Source	Sum of Squares	Df	Mean Square	*F*-Value	*p*-Value	Significance
Model	80.27	9	8.92	5.080	0.0217	*
A	1.83	1	1.83	1.050	0.3406	
B	6.21	1	6.21	3.540	0.1019	
C	0.10	1	0.10	0.058	0.8170	
A^2^	8.95	1	8.950	5.1000	0.0584	
B^2^	51.53	1	51.530	29.3800	0.0010	***
C^2^	0.43	1	0.430	0.2500	0.6345	
AB	1.07	1	1.070	0.6100	0.4601	
AC	2.34	1	2.340	1.3300	0.2859	
BC	4.28	1	4.280	2.4400	0.1620	
Residual	12.28	7	1.75			
Lack of fit	8.19	3	2.7300	2.6700	0.1832	
Pure error	4.09	4	1.02			
Cor total	92.54	16				
R^2^ = 0.9373						
R^2^_Adj_ = 0.9126						

*** means extreme significance of the difference (*p* < 0.001). * means significance of the difference (*p* < 0.05). Df means degree of freedom.

## Data Availability

Not applicable.

## Sample Availability

Samples of the compounds are definitely available from the authors.
